# Comparing the ability of the IAT and of the SC-IAT to account for behavioral outcomes: a re-analysis using linear mixed-effects models

**DOI:** 10.3389/fpsyg.2025.1652403

**Published:** 2025-10-29

**Authors:** Ottavia M. Epifania, Pasquale Anselmi, Egidio Robusto

**Affiliations:** 1 Department of Psychology and Cognitive Science, University of Trento, Trento, Italy; 2 Psicostat, University of Padova, Padova, Italy; 3 Department of Philosophy, Sociology, Education and Applied Psychology, Padova, Italy

**Keywords:** Rasch model, log-normal model, implicit association test, single category implicit association test, re-analyses

## Abstract

**Introduction:**

Implicit measures are widely used to indirectly assess psychological constructs and predict behavior. Nonetheless, comparisons of their predictive validity often suffer from methodological limitations, including administration inconsistencies, scoring differences, and unaccounted sources of variability related to data structure and experimental design.

**Methods:**

To address these issues, the present study re-analyzes an existing dataset comparing the Implicit Association Test (IAT) and its single-category variant (SC-IAT) using a modeling framework that integrates a Rasch-like parameterization of accuracies and response times while accounting for the fully crossed data structure and the within-subject design.

**Results:**

Results partially align with the original findings and further corroborate the higher predictive validity of the IAT, while revealing the specific contribution of one SC-IAT that was likely obscured in traditional scoring approaches.

## 1 Introduction

Implicit measures can be used for the indirect assessment of people's attitudes, opinions, and preferences from their responses to speeded categorization tasks (Greenwald and Lai, 2020), in an ever wider range of application fields (see Epifania et al., 2022b, for a review on the topic). A common application of implicit measures pertains the prediction of people's behavior, with several studies comparing their predictive power (e.g., Epifania et al., 2022a, 2023; Meissner et al., 2019; Perugini, 2005; Richetin et al., 2019). The predictive power of implicit measures refers to the extent to which person-level scores derived from implicit measures account for the variance in external variables, such as behavioral outcomes or explicit evaluations, within the same sample. These comparisons may be compromised by differences in the administration and scoring of the measures. More importantly, they may be affected by sources of error variance related to the structure of the data (i.e., fully-crossed design; Epifania et al., 2024; Westfall et al., 2014) and the within-subjects designs often used in such studies (e.g., Judd et al., 2012, 2017). Therefore, conclusions about the predictive power of implicit measures should be interpreted with caution.

In an attempt to gain a better understanding of the issue, Epifania et al. (2020a) compared the predictive power of two commonly used implicit measures, the implicit association test (IAT; Greenwald et al., 1998) and its single category variant, the single category IAT (SC-IAT; Karpinski and Steinman, 2006), by reducing as much as possible the administration differences and by introducing new scoring methods that allows for a greater comparability between the performance of the two measures. Their results suggested that the IAT has an overall better predictive validity than the SC-IAT. However, the authors overlooked the sources of variability in the data due to both the fully-crossed structure of the implicit measures and the within-subjects design, making the results difficult to interpret and dependent on the performed statistical analysis. Indeed, different analytical approaches applied to the same dataset to answer the same research questions might lead to different and even contrasting results (e.g., Silberzahn et al., 2018). This variability arises because each analytical strategy brings its own set of assumptions, model structures, and inferential frameworks, all of which can influence the interpretation of the underlying data patterns. As such, the choice of method can shape the conclusions drawn from the data, which, in this case, might wrongfully favor an implicit measure over the other.

In this contribution, we propose a re-analysis of the data from Epifania et al. (2020a) with the modeling approach for implicit measures introduced in Epifania et al. (2020d). This modeling framework incorporates a Rasch-like parameterization of accuracies and response times while controlling for the fully-crossed structure of implicit measures. Previous applications have shown promising results in predicting behavior (Epifania et al., 2022a) and in identifying administration features that influence respondents' performance (Epifania et al., 2023). In this contribution, the modeling approach is extended to account for the variability ascribable to the within-subjects experimental design used in Epifania et al. (2020a). By concurrently accounting for the sources of variability due to both the fully-crossed structures of implicit measures and to the within-subjects design, more robust inferences on the predictive validity of the IAT and the SC-IAT should be possible.

The manuscript is organized as follows. The next section presents the IAT, the SC-IAT, and their fully-crossed structure. Then, Rasch and log-normal models and their relationship with (generalized) linear (mixed-effects) models are briefly described. The specification of models with different random structures follows. The application of the modeling approach is illustrated, along with the comparisons between the predictive power of the typical scoring methods and the estimates obtained from their application. Some final remarks conclude the argumentation.

## 2 Implicit association test and single category-implicit association test

**Figure 1:**
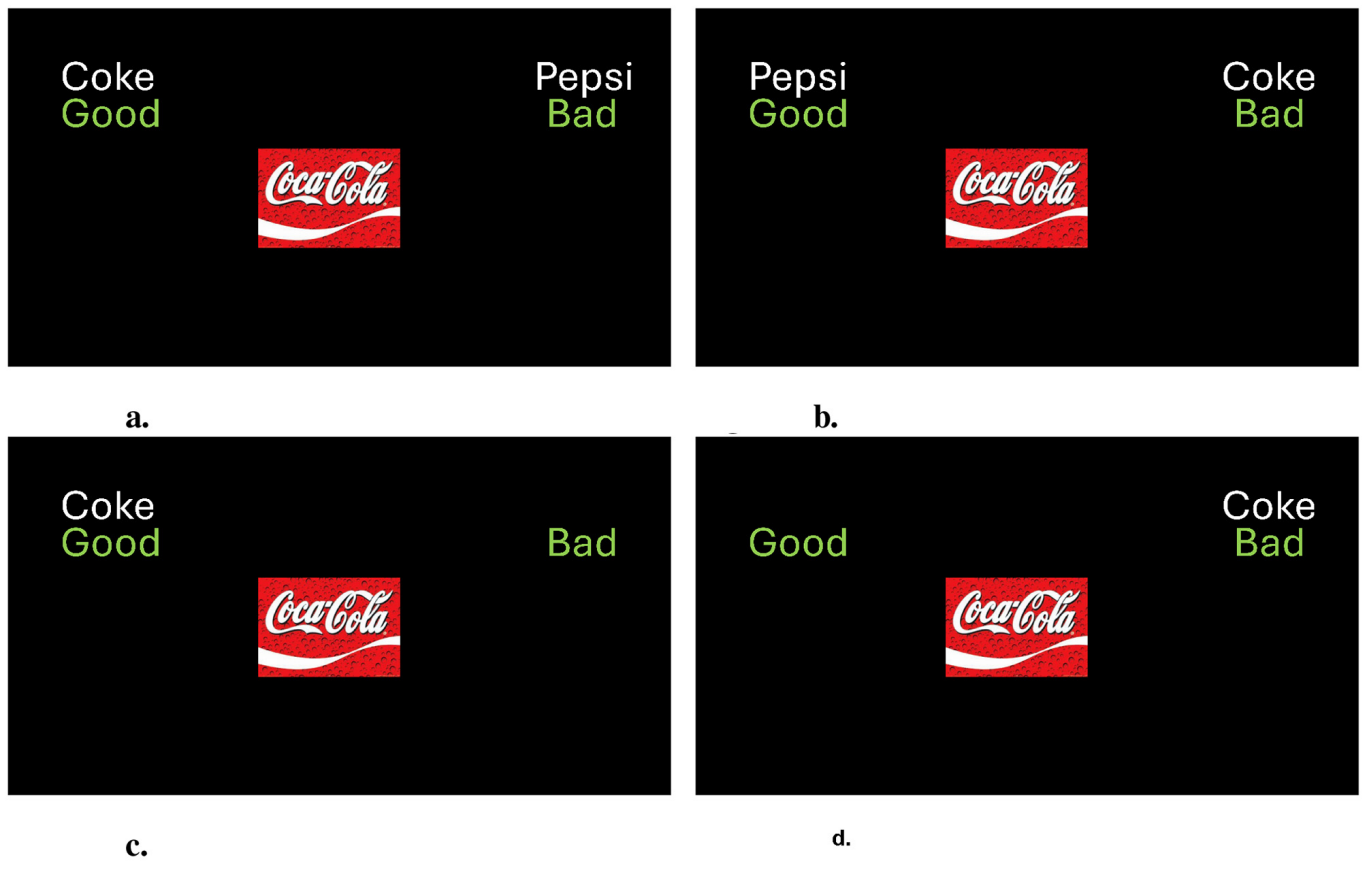
Associative conditions: **(a)** Coke-Good/Pepsi-Bad condition of a Soda IAT, **(b)** Coke-Bad/Pepsi-Good condition of a Soda IAT, **(c)** Coke-Good condition of a Coke SC-IAT, and **(d)** Coke-Bad condition of a Coke SC-IAT.

The IAT and the SC-IAT measure the strength of the associations between targets (e.g., *Coke* and *Pepsi* in a Soda IAT, *Coke* in a Coke SC-IAT) and evaluative dimensions (*Good* and *Bad*) by considering the speed and accuracy with which prototypical exemplars of targets or evaluative dimensions are sorted in their own category in two contrasting associative conditions. These exemplars (i.e., stimuli) appear one at a time on the computer screen and are categorized using two response keys located on the left and right sides of the keyboard. In one associative condition of the IAT (i.e., Coke-Good/Pepsi-Bad condition, Figure 1a, *CGPB*), *Coke* and *Good* exemplars share one response key, while *Pepsi* and *Bad* exemplars share the opposite key. In the contrasting condition (i.e., Pepsi-Good/Coke-Bad condition, Figure 1b, *PGCB*), *Pepsi* and *Bad* are assigned with one key, and *Coke* and *Bad* exemplars with the other. The SC-IAT employs a categorization task that closely resembles that of the standard IAT, but only the exemplars from one target category are presented. For instance, in one associative condition of a Coke SC-IAT (i.e., Coke-Good condition, Figure 1c), *Coke* and *Good* exemplars are assigned to the same response key, while *Bad* exemplars are assigned to the opposite key. In the contrasting associative condition (i.e., Coke-Bad condition, Figure 1d), *Coke* and *Bad* share one response key, while *Good* exemplars are assigned to the opposite key.

While the IAT provides a “comparative” measure of how much one of the targets is preferred over the other, the SC-IAT provides an “absolute” measure of how much a single target is positively (or negatively) evaluated. The IAT effect refers to the difference in the performance of the respondents between the associative conditions. Typically, the strength and direction of the IAT effect are quantified using *ad hoc* effect size measures known as *D* scores, which are computed as the standardized difference in average response times across trials between the two conditions (Greenwald et al., 2003; Karpinski and Steinman, 2006).

### 2.1 Fully-crossed design: challenges and (possible) solutions

In experiments with fully-crossed data design (e.g., Epifania et al., 2024; Westfall et al., 2014; Wolsiefer et al., 2017), such as the IAT and the SC-IAT, the same set of stimuli representing the superordinate categories is sorted by the respondents according to different rules in two contrasting conditions. The underlying assumption is that one of the conditions (i.e., the one that is consistent with the automatic association of the respondent) is “easier” than the other, in terms of faster and more accurate responses. Besides being typical of implicit measures, this structure can also be found in experiments pertaining psycholinguistics (e.g., Barr et al., 2013) or cognitive psychology (e.g., Vicovaro and Dalmaso, 2021).

Table 1 illustrates an example of a fully-crossed structure resulting from a simplified version of a Soda IAT, where stimuli representing the two target categories (*Coke* and *Pepsi*) and the two evaluative dimensions (*Good* and *Bad*) are administered in the two contrasting associative condition, denoted as *CGPB* and *PGCB*. If one of the two target categories of the table is dropped, the fully-crossed structure of the SC-IAT is obtained.

**Table 1:** Example of a fully-crossed structure where stimuli representing either target categories (*Coke* and *Pepsi*) or evaluative dimensions (*Good* and *Bad*) are administered in two contrasting associative conditions (*CGPB* and *PGCB*).

**Participants**	**Condition**	**Target categories**		**Evaluative dimensions**	
		**Coke1**	**Pepsi1**	**Evil**	**Laughter**
*p*_1_	*CGPB*	*T*_1, Coke1, *CGPB*_	*T*_1, Pepsi1, *CGPB*_	*T*_1, Evil, *CGPB*_	*T*_1, Laughter, *CGPB*_
	*PGCB*	*T*_1, Coke1, *PGCB*_	*T*_1, Pepsi1, *PGCB*_	*T*_1, Evil, *PGCB*_	*T*_1, Laughter, *PGCB*_
*p*_2_	*CGPB*	*T*_2, Coke1, *CGPB*_	*T*_2, Pepsi1, *CGPB*_	*T*_2, Evil, *CGPB*_	*T*_2, Laughter, *CGPB*_
	*PGCB*	*T*_2, Coke1, *PGCB*_	*T*_2, Pepsi1, *PGCB*_	*T*_2, Evil, *PGCB*_	*T*_2, Laughter, *PGCB*_
*p*_3_	*CGPB*	*T*_3, Coke1, *CGPB*_	*T*_3, Pepsi1, *CGPB*_	*T*_3, Evil, *CGPB*_	*T*_3, Laughter, *CGPB*_
	*PGCB*	*T*_3, Coke1, *PGCB*_	*T*_3, Pepsi1, *PGCB*_	*T*_3, Evil, *PGCB*_	*T*_3, Laughter, *PGCB*_

*CGPB* and *PBCG* represent the two contrasting conditions. Coke1 and Pepsi1 are example stimuli belonging to the Coke and Pepsi target categories, respectively, while Evil and Laughter are example stimuli from the Bad and Good evaluative dimensions. Stimuli can be administered in random order and may appear more than once within each condition. The order of conditions can be counterbalanced across respondents. *T* represents the trial that is obtained from the unique combination Respondent × Stimulus × Condition.

In a multilevel modeling perspective, the contrasting conditions (*CGPB* and *PBCG*) are at the highest level, including both respondents (*p*_1_, *p*_2_, *p*_3_) and stimuli (Coke1, Pepsi1, Evil, Laughter), while respondents and stimuli are at a same, lower level (e.g., Judd et al., 2012). The trials *T* are at the lowest level of observation (the cells in the table), resulting from the crossing between the respondents, the stimuli, and the conditions, hence representing a unique combination Respondent × Stimulus × Condition (Epifania et al., 2024). Being presented to the same respondents within and between conditions, the stimuli are crossed with both the conditions and the respondents. The same logic applies to the respondents, who are crossed with both the stimuli and the conditions.

The fully-crossed structure of implicit measures introduces dependencies among observations that must be accounted for in order to obtain reliable estimates and avoid biased results (e.g., Barr et al., 2013; Judd et al., 2012, 2017; McCullagh and Nelder, 1989; Westfall et al., 2014; Wolsiefer et al., 2017). However, data from tasks such as the IAT and the SC-IAT are usually analyzed with a by-participant approach that overlooks the crossing of respondents, stimuli, and conditions, and the variability associated with these levels and their crossing. In this approach, person-level scores are obtained by averaging responses across trials within conditions and then computing their difference (see, e.g., Raaijmakers, 2003). These scores are subsequently related to external variables in further analyses. Although straightforward, this scoring procedure implicitly assumes that all stimuli have the same effect on all participants, while also overlooking the variability related with the crossing of stimuli and respondents within and between conditions. As a consequence, error variance due to unmodeled stimulus- and respondent-level variability is absorbed into the person-level scores, which can obscure the effect of the associative conditions and yield biased or inconclusive inferences (Barr et al., 2013; Baayen et al., 2008; Judd et al., 2012, 2017; Raaijmakers, 2003; Wolsiefer et al., 2017; Westfall et al., 2014). These dependency issues are exacerbated in within-subject designs where multiple implicit measures are administered. In such cases, additional sources of variability – differences between measures at both respondent and stimulus levels – remain uncontrolled if each measure is analyzed in isolation. Linear mixed-effects models (LMMs) provide an ideal framework for dealing with both issues, since they allow for concurrently handling the dependencies related to both the fully-crossed structure of the implicit measures and the within-subject administration design (e.g., Epifania et al., 2024; Judd et al., 2012).

Besides accounting for the sources of variability due to the fully-crossed design of implicit measures, the modeling framework based on LMMs and Generalized LMMs (GLMMs) in Epifania et al. (2020d) provides a Rasch-like parameterization of both accuracy and response times. This approach allows for a deeper understanding of implicit measures functioning (e.g., by isolating each stimulus contribution to the overall effect) and for deriving more reliable person-level estimates. When compared with typical scoring methods of implicit measures, the person-level estimates obtained with the modeling approach yield more accurate predictions of behavior (e.g., Epifania et al., 2022a, 2023). This contribution applies and extend the modeling approach to instances where different implicit measures are administered concurrently to the same sample of respondents while employing the same set of stimuli across measures (i.e., within-subjects design). The person-level estimates are compared against the usual scoring methods to investigate the predictive validity of an IAT for the investigation of the preference for dark or milk chocolate (denoted as Chocolate IAT) and its single categories variants (denoted as Dark SC-IAT and Milk SC-IAT, respectively).

## 3 Rasch, log-normal, and (generalized) linear models

According to the Rasch model (Rasch, 1960), the probability of observing a correct repsonse to stimulus *s* by respondent *p* depends on both the respondent's ability (i.e., as described by the ability parameter θ_*p*_) and the stimulus difficulty (i.e., as described by the difficulty parameter *b*_*s*_). The Rasch model corresponds to a generalized linear model (GLM) with a logit link function that relates the linear combination of predictors to the binomially distributed responses. Accordingly, a Rasch-like parameterization of accuracies can be obtained by applying a GLM with a logit link function (e.g., De Boeck et al., 2011; Doran et al., 2007). However, when a GLM is applied to obtain a Rasch-like parametrization of the data, the interpretation of the stimulus parameter *b*_*s*_ is reversed, such that it is interpreted as an *easiness* parameter (i.e., the higher the value of *b*_*s*_, the easier stimulus *s* is) (e.g., Doran et al., 2007). In what follows, the stimulus parameter obtained by means of the GLM will be referred to as easiness parameter.

The log-normal model (van der Linden, 2006) allows for interpreting the observed log-time response as a function of the speed with which the person responds (i.e., the speed parameter τ_*p*_, the higher the value, the faster *p* is) and the time each stimulus requires to get a response (i.e., time intensity parameter δ_*s*_, the higher the value, the longer *s* requires to get a response). This model provides a Rasch-like parametrization of log-transformed response times. By applying a linear model (LM) with an identity link function to the log-transformed response times, it is possible to obtain log-normal-like estimates of the log-transformed response times (e.g., Epifania et al., 2020d, 2024). When a LM is applied to the log-transformed response times, the interpretation of the speed parameter τ_*p*_ is reversed, such that the higher the value of τ_*p*_ the slower respondent *p*. Nonetheless, in what follows we will be referring to the τ parameter as speed parameter.

To account for the fully-crossed structures and the within-subjects experimental design, the linear combination of predictors in the (G)LM needs to be extended to include the random effects related to the respondents, the stimuli, the implicit measures, and the associative conditions within the implicit measures (Epifania et al., 2024). The distribution of the random effects is estimated as a multivariate normal distribution (i.e., MVN) with mean 0 and a variance-covariance matrix **Σ** that is determined by the vector of parameters of the random effects Γ (Doran et al., 2007). The dimension of Γ is usually rather small, and its size derives from the number of random factors specified in the model, regardless of the number of levels they include. The Rasch-like estimates are derived by combining the estimates of the fixed effects with the *best linear unbiased predictors*, (BLUPs Doran et al., 2007). The BLUPs describe the deviation of each level of the random effects from the fixed effects. As such, person-level estimates are influenced by the specific random-effects structure used in the model, which is designed to capture the variability present in the data. Differently from the usual scoring methods of implicit measures that concurrently account for the response times and the accuracies (the error responses are given a penalty), this modeling approach separately considers the information that can be gathered from the accuracy performance and the time performance of the respondents. The person-levels estimates of the best fitting models are used for predicting the behavioral choice and their performance is compared against that of the typical scoring methods.

### 3.1 Fixed and random structures of (G)LMMs

Potentially, the specification of the random structure in (G)LMMs can account for all possible sources of variability associated with both the fully-crossed structure of the data and the within-subjects design (i.e., maximal models; Barr et al., 2013). Nonetheless, Bates et al. (2015a) cautioned that overly complex random structures are at risk of convergence failure, because the data often do not provide sufficient information to estimate all parameters reliably. In practice, this means that while maximal models are theoretically appealing, they may not yield actual gains in model fit or inference if the complexity exceeds what the observed variability can support. As such, only the random structures that are meaningful for obtaining Rasch-like parametrizations of response accuracies and log-times are here presented. Additionally, since our primary focus is on person-level estimates, none of the random structures models within-stimulus variability, either between conditions or across implicit measures. In other words, stimuli are always modeled as random intercepts.

In all models, the fixed intercept is set to 0, such that none of the levels of the fixed slope is taken as the reference category and the estimates of each level of the fixed effects can be interpreted as the marginal mean in each of the levels. An overview of the linear combination of predictors η_*ps*_, including both fixed and random effects, is reported in Table 2, as well as the basic notation to fit the models in the lme4 package (Bates et al., 2015b) in R (R Core Team, 2018).

**Table 2:** Overview of the model structures and lme4 notation.

**Model**	**Linear predictor η**	**Respondents random factors**	**lme4 notation**
1	η_*ps*_ = α+β_*m*_*X*_*m*_+α_[*s*]_+α_[*p*]_	$\alpha_{\left[p\right]}\sim N\left(0,\sigma_{P}^{2}\right)$	~ 0 + measure + (1|stimuli) + (1|respondents)
2	η_*ps*_ = α+β_*m*_*X*_*m*_+α_[*s*]_+β_*m*[*p*]_*X*_*m*_	$\beta_{m\left[p\right]}\sim \mathrm{MVN}\left(0,\Sigma_{M}\right)$	~ 0 + measure + (1|stimuli) + (0 + measure|respondents)
3	η_*ps*_ = α+β_*c*_*X*_*c*_+α_[*s*]_+β_*c*[*p*]_*X*_*c*_	$\beta_{c\left[p\right]}\sim \mathrm{MVN}\left(0,\Sigma_{C}\right)$	~ 0 + condition + (1|stimuli) + (0 + condition|respondents)

*p* = 1, …, *P*: Respondent, *s* = 1, …, *S* Stimulus, *m* = 1, …, *M*: Implicit measure, namely IAT, Dark SC-IAT, Milk SC-IAT, *c* = 1, …, *C* Associative condition within each implicit measure, namely: IAT - MGDB, IAT - DGMB, Dark SC-IAT DG, Dark SC-IAT DB, Milk SC-IAT MG, Milk SC-IAT MB. Variables *X*_*m*_ and *X*_*c*_ are dummy variables. The dependent variable *y* can be either the response accuracies (GLMMs) or the log-time responses (LMMs). The fixed intercept α is set to 0. By doing so, the estimates of the fixed effects in Models 1 and 2 can be interpreted as either the log-odds of the probability of responding correctly (accuracy models) or the expected average log-time responses (log-time models) in each of the implicit measures, while those in Model 3 are the expected same values but considering each condition of each implicit measure. N identifies a normal distribution, MVN identifies a multivariate normal distribution, defined by the variance-covariance matrix Σ. In all models, the random effects of the stimuli are $\alpha_{s}\sim N\left(0,\sigma_{S}\right)$. The notations (1|stimuli) and (1|respondents) are used to specify the random intercepts of the stimuli and of respondents, respectively. The notations (0 + measure|respondents) and (0 + condition|respondents) are used to specify the random slopes of the respondents in *M* (Model 2) and in *C* (Model 3).

The differences between the GLMMs applied to accuracies and the LMMs applied to log-time responses concern: (i) the dependent variable *y*, either the accuracy response (0—incorrect response vs. 1—correct response) or the log-transformed response time to each trial resulting from the unique crossing respondent × stimulus × associative condition nested within each implicit measure; and (ii) the function used to link the linear combination of predictors to the observed response, either a logit link function in the GLMMs or an identity function in the LMMs. Moreover, the response accuracy in the GLMMs is modeled with the logistic link as conditioned to the random structure, such that no residual error term ε is included. On the other hand, in the LMMs the residual error is assumed to follow a normal distribution, $\varepsilon \sim N\left(0,\sigma^{2}\right)$. In the empirical application that follows, the models applied to the accuracies will be denoted with letter A, while those applied to the log-time responses will be denoted with letter T.

In all models, the stimuli are modeled as random intercepts (α_[*s*]_) to account for between–stimulus variability across implicit measures (Models 1 and 2) or across associative conditions within implicit measures (Model 3). The estimates of the stimulus parameters, either *b*_*s*_ (GLMMs) or δ_*s*_ (LMMs), derive from their random effects $\alpha_{\left[s\right]}\sim N\left(0,\sigma_{S}^{2}\right)$. Given this specification, overall stimuli estimates are obtained from all models. The estimates of person parameters, either θ_*p*_ (GLMMs) or τ_*p*_ (LMMs), derive from the random effects of the respondents (column “Respondent random factors” in Table 2), and vary according to their specification in the models. Considering the intercepts of the respondents α_[*p*]_ ($\alpha_{\left[p\right]}\sim N\left(0,\sigma_{P}^{2}\right)$), Model 1 addresses the between–respondent variability across implicit measures. Model 1 provides overall respondent estimates across implicit measures, either θ_*p*_ or τ_*p*_. This model is expected to be the best fitting one when low variability at both respondent and stimulus levels is observed, suggesting that neither the performance of the respondents nor the functioning of the stimuli change between implicit measures and associative conditions. Considering the random slopes of respondents in implicit measures β_*m*[*p*]_ ($\beta_{m\left[p\right]}\sim \mathrm{MVN}\left(0,\Sigma_{M}\right)$), Model 2 addresses the within–respondents within – measures variability and results in measure–specific respondent estimates, either θ_*m*[*p*]_ or τ_*m*[*p*]_. This model is expected to be the best fitting one when high within–respondents between–measures variability is observed, suggesting that the performance of the respondents changes between implicit measures. In Model 3, the random slopes of respondents in the associative conditions within each implicit measure β_*c*[*p*]_ ($\beta_{c\left[p\right]}\sim \mathrm{MVN}\left(0,\Sigma_{C}\right)$) are specified to account for the within–respondents between–conditions variability. This model provides condition–specific respondent estimates within each implicit measure, either θ_*c*[*p*]_ or τ_*c*[*p*]_. Model 3 is expected to be the best fitting model when high within–respondents variability between associative conditions of each implicit measure is observed, suggesting that their performance is affected by the associative condition of each implicit measure. The difference between condition–specific estimates expresses the bias on the performance of the respondents due to the associative conditions in each implicit measure.

The best fitting model for accuracies and log-time responses was determined by independently comparing candidate models according to the Akaike Information Criterion (AIC, AIC=-2log(L)+2k; Akaike, 1974) and the Bayesian Information Criterion (BIC, BIC=-2log(L)+klog(n); Schwarz, 1978). Both AIC and BIC are entropy indexes based on the log-likelihood L of the models, which is penalized according to the number of *k* parameters included in the model. Moreover, the BIC adds a penalty related to the sample size *n*, such that complex models receive a stronger penalty as the sample size increases. When the two criteria favor different models, the AIC typically points toward the one with higher predictive accuracy, while the BIC tends to select a more parsimonious solution, aiming to approximate the “true” underlying model. In this sense, AIC is more suitable when the priority is prediction, while BIC is more appropriate when the goal is theoretical parsimony (e.g., Zhang et al., 2023). In this application, we considered both indexes, but AIC was favored in the event of disagreement. The reader interested in the counting of the free parameters *k* that are used for the AIC and BIC penalization can refer to Epifania et al. (2024).

## 4 Method

A Chocolate IAT, a Milk chocolate SC-IAT, and a Dark chocolate SC-IAT were used in the original study in Epifania et al. (2020a). The models used in this manuscript to re-analyze the data were fitted in R R Core Team (2018) with the lme4 package Bates et al. (2015b) (bobyqa optimizer). The implicitMeasures package Epifania et al. (2020c, 2025) was used for computing IAT and SC-IAT *D* scores. The IAT *D* score can also be computed with the DScoreApp Epifania et al. (2020b). Graphical representations were obtained with ggplot2 Wickham (2016).

### 4.1 Participants

The sample of the original study in Epifania et al. (2020a) was composed of 152 people (*F* = 63.55%, Age = 23.95 ± 2.83 years), recruited at the University of Padova. Majority of the participants were students (94.08%).

### 4.2 Materials and procedure

Twenty-six attributes were used to represent the two evaluative dimensions *Good* and *Bad* (13 exemplars for each of them) and fourteen chocolate images were used to represent the two targets *Dark* and *Milk* (seven for each of them).

Dark and milk chocolate images were presented in the Chocolate IAT. The critical blocks were composed of 60 trials each (Greenwald et al., 2003), defining the Dark-Good/Milk-Bad condition (DGMB), and the Milk-Good/Dark-Bad condition (MGDB). The SC-IATs employed only either dark (Dark SC-IAT) or milk (Milk SC-IAT) chocolate images. The critical blocks of the SC-IATs were composed of 72 trials each (Karpinski and Steinman, 2006). The critical blocks of the dark SC-IAT were the Dark-Good/Bad (DG) condition and the Good/Dark-Bad (DB) condition. The critical blocks of the milk SC-IAT were the Milk-Good/Bad (MG) condition and the Good/Milk-Bad (MB) condition.

After completing the tasks, the experimenter offered a free chocolate bar—either dark or milk chocolate—to each respondent as a token for their participation. The choice was recorded after the participants had left the laboratory.

The original study included the explicit assessment of the preference for dark or milk chocolate through Likert-type scales. The explicit assessments were used for supplementary analysis, where the incremental validity of the IAT and the two SC-IATs with respect to the prediction of the chocolate choice was investigated through hierarchical multiple logistic regressions. Results indicated that the measure provided by any of the implicit measures employed was useful for predicting the choice when the explicit evaluations were taken into account. This analysis is not relevant in the current study, and it was hence not included.

### 4.3 Data cleaning and *D* score computation

The IAT was scored with the *D4* algorithm in Greenwald et al. (2003) (i.e., trials > 10,000 ms were discarded, incorrect responses were replaced by the average response time inflated by a 600 ms penalty). Positive scores indicate a preference for dark chocolate over milk chocolate. The SC-IAT was scored according to Karpinski and Steinman (2006) (i.e., trials < 350 ms were discarded, incorrect responses were replaced by the average response time inflated by a 450 ms penalty). In both SC-IATs, positive scores indicate a positive evaluation of the target chocolate. The raw response times of each trial were used for estimating the log-normal model (i.e., the error latencies were not assigned any penalty).

In the original paper by Epifania et al. (2020a), the main objective was to examine how different strategies for handling outliers and applying error penalties influenced the robustness of the results. To ensure that these comparisons were meaningful, the authors also minimized procedural differences in how the implicit measures were administered. Their findings showed that the choice of scoring method did not substantially affect the outcomes. Building on this evidence, in the present study we adopted the standard scoring procedures for both the IAT and the SC-IAT. At the same time, our focus is on comparing these measures with results obtained through an alternative analytic approach, which is theoretically better equipped to account for both the fully crossed structure of the implicit measures data and the variability inherent in the within-subject experimental design.

### 4.4 Ability-based and speed-based measures of the IAT effect

This section provides an overview of the information offered by each model and of the person-level scores that can be computed from them.

While addressing the implicit measures and associative conditions variability, Model A3 and Model T3 (see Table 2) allow for obtaining condition–specific ability and speed estimates for each respondent, respectively. As such, the IAT effect can be investigated considering both the accuracy and time performance of the respondents in each implicit measure by computing ability-based and speed-based differential measures. However, this investigation is possible if Model 3 is the best fitting model on both accuracies and time responses.

If Model A3 results as the best fitting model on accuracy responses, ability-based differential measures are computed such that a positive score indicates: (i) in the IAT, higher ability in the DGMB condition than in the MGDB one, (ii) in the Dark SC-IAT, higher ability in the DG rather than DB condition, and (iii) in the Milk SC-IAT, higher ability in the MG rather than MB condition. If Model T3 results as the best fitting model on accuracy responses, speed-based differential measures are computed such that a positive score indicates: (i) in the IAT, higher speed in DGMB rather than in MGDB condition, (ii) in the Dark SC-IAT, higher speed in DB rather than in DG condition, and (iii) in the Milk SC-IAT, higher speed in MB rather than in MG condition.

If Model 2 or Model 1 result as the best fitting model, either the measure-specific or overall estimates of the respondents are obtained.

### 4.5 Logistic Models for the prediction of the behavioral outcome

If Models A3 and T3 are the best fitting ones, the ability-based and speed-based differential measures of the IAT effect can be used to predict the behavioral outcome. Additionally, their predictive power can be compared against that of the typical scoring methods. Besides considering the ability-based and speed-based differential measures and the typical scoring methods, the linear combination of their respective single components are used to predict the behavioral choice. The single components of the ability-based and speed-based differential measures are the condition–specific ability and condition–specific speed estimates, respectively, while those of the typical scoring methods are the average response times in each associative condition of the implicit measures. Such an analysis would allow for disentangling the preference mostly involved in the behavioral choice. Moreover, it has been pointed out that using differential measures to express the bias due to the associative conditions of the IAT can lead to unreliable results due the strong assumptions on which such measures rely (see, e.g., Fiedler et al., 2006).

In such a scenario, four models can be specified considering different predictors: (i) linear combination of the typical scoring methods of each implicit measure, (ii) linear combination of the condition–specific average response times of each implicit measure (i.e., single components of the typical scoring methods), (iii) linear combination of the ability-based and speed-based differential measures obtained from the model estimates for each implicit measure, and (iv) linear combination of the condition–specific ability and speed estimates of each implicit measure (i.e., single components of the model estimates). The selection of relevant predictors can be achieved using stepwise regression techniques. In this case, a forward selection approach is employed, starting with a null model that includes only the intercept as the baseline. This null model serves as a reference point against which all potential predictors are evaluated. Predictors are then sequentially added to the model based on their statistical significance and contribution to improving the model's explanatory power. *Nagelkerke's R*^2^ Nagelkerke (1991) was computed as *Pseudo R*^2^.

To further understand the combination of predictors that best accounts for the choice, the following statistics are computed: (i) proportion of choices correctly identified by the model (model general accuracy of prediction), (ii) proportion of dark chocolate choices (DCCs) correctly identified by the model (DCCs accuracy), and (iii) proportion of milk chocolate choices (MCCs) correctly identified by the model (MCCs accuracy). The MCC was coded as 1 and the DCC was coded as 0 in the data.

## 5 Results

### 5.1 Model comparison

The results of the models fitted on the accuracies and log-time responses are reported in Table 3.

**Table 3:** Results of the model comparison between the models fitted on the accuracies (A) and the log-time responses (T).

**Predictors**	**A1**	**A2**	**A3**	**T1**	**T2**	**T3**
IAT	3.27 (0.08)	3.26 (0.09)		−0.24 (0.02)	−0.24 (0.02)	
Dark SC-IAT	3.21 (0.08)	3.27 (0.09)		−0.46 (0.02)	−0.46 (0.02)	
Milk SC-IAT	3.20 (0.08)	3.25 (0.09)		−0.47 (0.02)	−0.47 (0.01)	
IAT—DGMB			2.92 (0.09)			−0.12 (0.02)
IAT—MGDB			4.05 (0.13)			−0.36 (0.02)
Dark SC-IAT—DB			3.45 (0.11)			−0.47 (0.02)
Dark SC-IAT—DG			3.23 (0.10)			−0.45 (0.02)
Milk SC-IAT—MB			3.22 (0.10)			−0.45 (0.02)
Milk SC-IAT—MG			3.42 (0.10)			−0.50 (0.01)
Observations	62,013	62,013	62,013	62,013	62,013	62,013
Log likelihood	−11,490.420	−11,442.900	−11,154.430	−22,305.840	−21,713.150	−19,437.270
AIC	22,990.850	22,905.800	22,364.850	44,623.690	43,448.310	38,932.540
BIC	23,036.020	22,996.150	22,617.830	44,677.900	43,547.700	39,194.560

The columns in gray indicate the best fitting accuracy and response time models according to model comparison. The standard errors are reported between parentheses. DGMB: Dark/Good-Milk/Bad condition of the IAT, MGDB: Milk/Good-Dark/Bad condition of the IAT, DG: Dark/Good condition of the Dark SC-IAT, DB: Dark/Bad condition of th Dark SC-IAT, MG: Milk/Good condition of th Milk SC-IAT, MB: Milk/Bad condition of the Milk SC-IAT. All *p*-values associated to the coefficients of the fixed effects of the GLMMs were < 0.001. The *p*-values associated to the coefficients of the fixed effects in LMMs are not reported because of the difficulty in accurately determining the degrees of freedom required for computing the critical *t*-values (see, e.g., Epifania et al., 2024).

Model A3 and Model T3 (i.e., the ones addressing the within–respondent between–condition and implicit measure variability) showed the least AIC and BIC on both accuracies and log-time responses, hence resulting as the best fitting ones. These models allow for the estimation of condition–specific ability (θ_DGMB[*p*]_, θ_MGDB[*p*]_, θ_DG[*p*]_, θ_DB[*p*]_, θ_MG[*p*]_, θ_MB[*p*]_) and speed (τ_DGMB[*p*]_, τ_MGDB[*p*]_, τ_DG[*p*]_, τ_DB[*p*]_, τ_MG[*p*]_, τ_MB[*p*]_) parameters. As such, ability-based and speed-based differential measures can be obtained for each implicit measure.

The results on the fixed effects of Model A3 indicate that the associative conditions where milk chocolate was associated with positive attributes and dark chocolate was associated with negative attributes (i.e., IAT—MGDB, Milk SC-IAT—MG, Dark SC-IAT—DB) tended to be easier than their respective counterparts (i.e., IAT—DGMB, Milk SC-IAT—MB, Dark SC-IAT—DG). Nonetheless, the results on the fixed effects of GLMMs should be taken with caution unless robust statistics are used to obtain the *p*-values (see e.g., Andreella et al., 2025). Similar results were observed on the log-time responses, such that responses tended to be faster when milk chocolate was associated with positive attributes and dark chocolate was associated with negative attributes than in the respective contrasting conditions. For both accuracies and log-transformed response times, greater variability between conditions was observed in the IAT compared to the SC-IAT, where the coefficients for the associative conditions were more similar. This suggests that the performance of the respondents is more strongly influenced by the associative conditions in the IAT than in the SC-IAT.

### 5.2 Prediction of the behavioral choice

**Figure 2:**
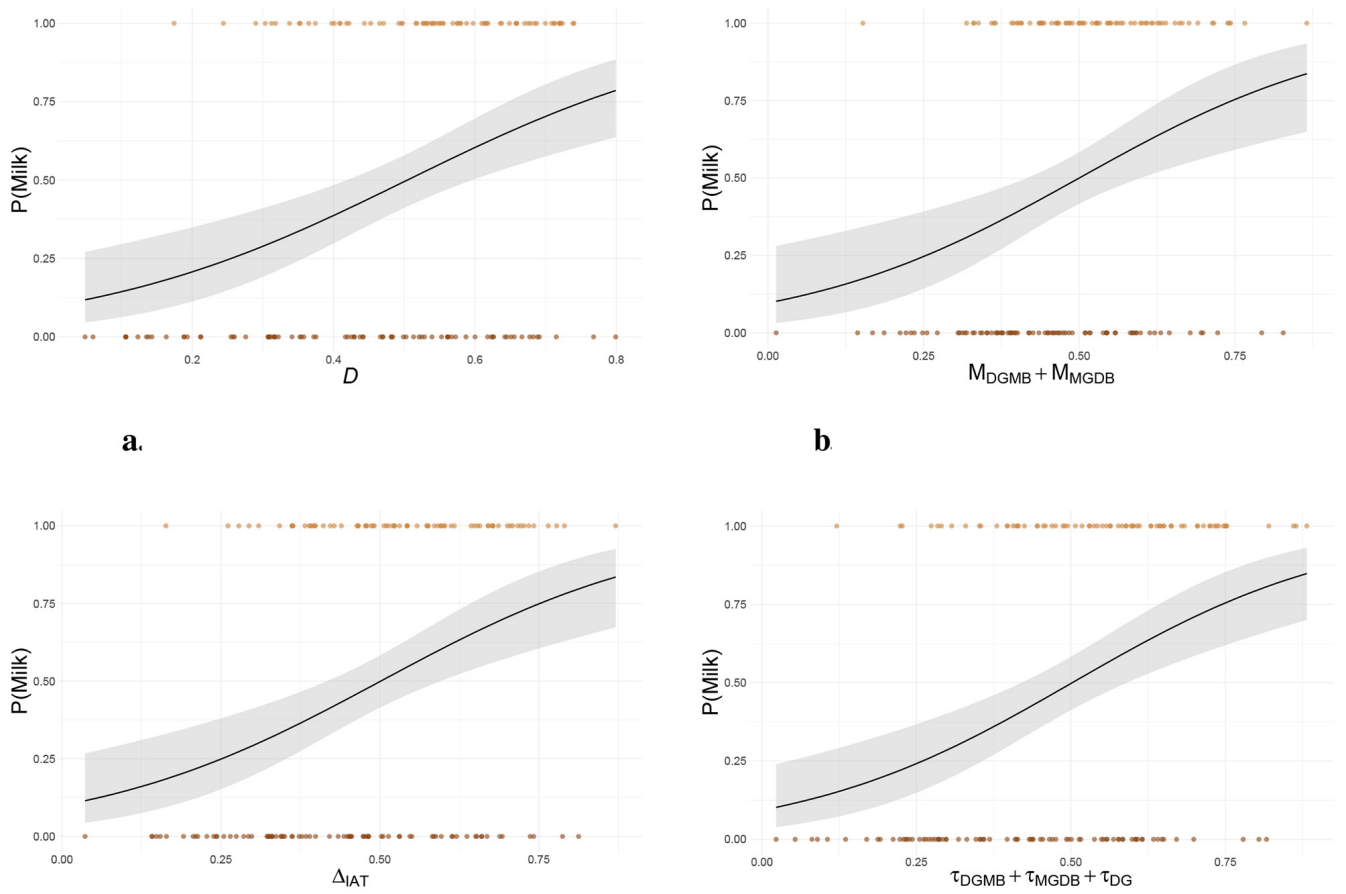
Predictors left in the models obtained with stepwise logistic regressions: **(a)** Model resulting from the linear combination of typical scoring methods, **(b)** Model resulting from the linear combination of the single components of typical scoring methods, **(c)** Model resulting from the linear combination of differential measures obtained from model estimates, and **(d)** Model resulting from the linear combination of the model estimates. DGMB: Dark/Good-Milk/Bad condition of the IAT, MGDB: Milk/Good-Dark/Bad condition of the IAT, DG: Dark/Good condition of the Dark SC-IAT, DB: Dark/Bad condition of the Dark SC-IAT, MG: Milk/Good condition of the Milk SC-IAT, MB: Milk/Bad condition of the Milk SC-IAT.

At the end of the experiment, the 48% of the respondents chose the milk chocolate bar. In the original study, participants were unaware of the availability of a chocolate bar during the experiment, as it was presented only at the very end, after all experimental procedures had been completed, as a token of participation. The chocolate bar was offered to all participants. Results of the models obtained with stepwise logistic regressions are illustrated in Figure 2.

All models explained about the same proportion of variance, ranging from 0.12 [Model (ii)] to 0.19 [Model (iv)]. Concerning the typical scoring methods, both differential measures (i.e., *D* score, model (i) in the figure) and single components [model (ii) in the figure] suggested that only the measures provided by the IAT are relevant for predicting the behavioral choice, ruling out the contribution of any of the two SC-IATs. Concerning the model accuracy of prediction, the models resulted in general accuracies of .64 (DCC = .62 and MCC = .67) and .62 (DCC = .67 and MCC = .56), respectively. Differential measures [model (iii) in the figure] suggested that only the speed-based measure of the IAT is relevant for predicting the choice. When the single components were taken into account [model (iv) in the figure], besides the condition–specific speed parameters of the IAT, also the speed parameter of the DG condition of the Dark SC-IAT was deemed relevant for the choice prediction. Importantly, all accuracy-based differential measures as well as the condition–specific ability estimates for each implicit measures were excluded from the linear combination of predictors, suggesting that they do not contribute to the choice prediction. Both model (iii) and model (iv) appeared to perform slightly better than model (i), suggesting that the model estimates can provide a better prediction of the behavioral choice than the *D* score. All models except model (ii) resulted in the same general accuracy (0.64), but achieved it through different combinations of MCC and DCC accuracy. Model (i) resulted in a DCC accuracy of 0.62 and in the highest MCC accuracy (0.67), while model (iii) resulted in the opposite instance (DCC = 0.67, MCC = 0.62). Finally, the DCC and MCC accuracies provided by model (iv) were 0.65 and 0.63, respectively.

## 6 Final remarks

Given the rising interest in the investigation of the robustness and stability of the results in experimental psychology (see, e.g., Silberzahn et al., 2018), this study presented the re-analysis of the data from Epifania et al. (2020a) with a modeling approach that, differently from the original study, allows for controlling the sources of variability related to the fully-crossed structure of the data and to the within-subjects experimental design. Investigating the predictive power of implicit measures with different statistical approaches can help in understanding which of the implicit measures is best able to predict behavioral outcomes, given a specific context. Although part of the results aligned with the ones in the original study, the presented approach highlighted the contribution of one the SC-IATs in the prediction of the behavioral choice, hence allowing for a deeper understanding of the choice behavior and of the processes underneath. This result allowed for speculating that the choice was mostly driven by the positive evaluation (i.e., preference) for dark chocolate than any association with milk chocolate. The contribution of the Dark SC-IAT was lost when typical scoring methods, differential measures, and single components of typical scoring methods were used for the prediction.

Considering the pattern of results observed in this study, different considerations arise. Firstly, given that the measure provided by the IAT is consistently relevant for predicting choice, regardless of whether single components, typical scoring methods, or differential measures are considered, it appears to be the implicit measure most strongly associated with choice behavior. At the same time, when the measures provided by the SC-IATs are analyzed with the appropriate modeling approach, they reveal additional processes involved in the choice, potentially leading to a better understanding on the processes involved in people's behaviors. Indeed, by aggregating across trials and ignoring the variability related to the fully-crossed structure, traditional scoring methods of implicit measures might include error variance components that can confound the effects of interest. By explicitly modeling this variability, the modeling framework proposed in this study allows for more precise estimation of person-level scores, potentially revealing patterns that standard scoring methods may miss. These findings might be of particular relevance for contexts in which implicit measures are used to shed light on behavior. For example, in intergroup research, implicit attitudes are often examined in relation to social decision-making (see Kurdi et al., 2019, for a meta-analysis). While our study focuses on consumer choice, the proposed approach to the analysis of implicit measures administered in a within-subjects design may potentially be adapted to examine whether decisions in intergroup contexts (e.g., affiliation with stigmatized groups) are more strongly driven by in-group favoritism or out-group derogation. Nonetheless, such applications remain speculative until validated in the specific domains pertaining intergroup relations. Moreover, implicit attitudes are often used in marketing research to predict consumer preferences and brand choices (e.g., Brunel et al., 2004). In these applications as well, the proposed modeling strategy may contribute to more precise insights, for instance in designing campaigns for specific targets.

Finally, the presented approach proved its usefulness for the investigation and comparison of the predictive ability of implicit measures administered in within-subjects designs. Moreover, the same approach is feasible for the analysis of other experiments with fully-crossed structure, such as those for the investigation of the SNARC effect (see, e.g., Epifania et al., 2024). In this light, future studies might employ the presented approach for analyzing and comparing other experiments with fully-crossed structures administered in within-subject designs.

## Data Availability

The original contributions presented in the study are included in the article/supplementary material, further inquiries can be directed to the corresponding author.
