# Correction: Comparing the ability of the IAT and of the SC-IAT to account for behavioral outcomes: a re-analysis using linear mixed-effects models

**DOI:** 10.3389/fpsyg.2025.1764815

**Published:** 2026-01-09

**Authors:** Ottavia M. Epifania, Pasquale Anselmi, Egidio Robusto

**Affiliations:** 1 Department of Psychology and Cognitive Science, University of Trento, Trento, Italy; 2 Psicostat, University of Padova, Padova, Italy; 3 Department of Philosophy, Sociology, Education and Applied Psychology, Padova, Italy

**Keywords:** Rasch model, log-normal model, implicit association test, single category implicit association test, re-analyses

The reference for **Epifania et al., 2024** was erroneously omitted from the final publication.

The reference appears in full below:

Epifania, O. M., Anselmi, P., and Robusto, E. (2024). A guided tutorial on linear mixed-effects models for the analysis of accuracies and response times in experiments with fully crossed design. *Psychol. Methods*. doi: 10.1037/met0000708

The original version of this article has been updated.

